# Comprehensive Analysis of Unsymmetrical Dimethylhydrazine: Adsorption Behavior, Environmental Fate, and Toxicity Across Contrasting Soil Matrices

**DOI:** 10.3390/toxics13100859

**Published:** 2025-10-11

**Authors:** Juan Du, Xianghong Ren, Yizhi Zeng, Lei Zhang, Jinfeng Shi, Shuai Yang

**Affiliations:** 1 Rocket Force University of Engineering, Xi’an 710025, China; renxh701@163.com (X.R.); 13201556440@163.com (Y.Z.); 18629522225@163.com (J.S.); yangshuai@chd.edu.cn (S.Y.); 2 State Key Laboratory of Coal-Based Clean Energy, Xi’an Thermal Power Research Institute Co., Ltd., Xi’an 710054, China; zhangleix@tpri.com.cn

**Keywords:** unsymmetrical dimethylhydrazine, adsorption behavior, environmental toxicity, soil matrix

## Abstract

Unsymmetrical dimethylhydrazine (1,1-Dimethylhydrazine, UDMH) is widely used as a high-performance liquid rocket propellant for the space industry globally. The release and leakage of UDMH into the environment, especially the soil environment, pose serious threats to ecosystems and human beings. In order to reveal the hazards of UDMH to soil and facilitate subsequent remediation, the adsorption behavior of UDMH in typical soil (yellow-brown soil, red soil, and black soil) matrices was explored, the environmental fate and toxicity of UDMH were presented by simulation calculation, and the phytotoxicity was evaluated by germination assay in the present study. The results showed that the adsorption performance of red soil, yellow-brown soil, and black soil for UDMH increased sequentially by integrating the findings from kinetic and thermodynamic studies. A highly significant correlation between the physicochemical and adsorption parameters for various soil matrices indicated a considerable impact of soil physicochemical properties on the adsorption behavior of UDMH in soils. The environmental fate simulation calculation indicated that UDMH and its transformation products were prone to being dissolved in soil water and migrating; however, once these compounds were present in the surface layer of dry soil, severe ecological and environmental pollution would occur. Based on a thorough evaluation of the toxicity parameters, formaldehyde dimethylhydrazone has been identified as demonstrating the most pronounced environmental toxicity profile, thus warranting prioritized attention. The results of a germination assay demonstrated that more than 100 mg·kg^−1^ of UDMH in the soil would lead to strong phytotoxicity to plants, and more than 200 mg·kg^−1^ of UDMH would significantly affect the early germination of seeds. Hence, this research provided helpful insights and theoretical support for the environmental fate and remediation of UDMH.

## 1. Introduction

Unsymmetrical dimethylhydrazine (1,1-Dimethylhydrazine, UDMH), a high-energy fuel, has been extensively employed as a propellant in the space industry globally [1,2,3,4,5]. The launch of space vehicles can lead to the release and leakage of UDMH into the environment, especially the soil environment, thereby posing significant threats to both ecosystems and human populations. UDMH is a highly hazardous substance with profound impacts on living organisms, and it can adversely affect soil biological activity [6,7,8,9,10]. Moreover, due to its high carcinogenicity, mutagenicity, teratogenicity, and embryotoxicity, the International Agency for Research on Cancer has classified UDMH as a possible human carcinogen (Group 2B). Meanwhile, the high reactivity of UDMH results in the rapid formation of a vast array of nitrogen-containing products due to its oxidative transformation or interaction with each other, which also possess toxicity [11,12,13,14,15]. The increasing usage of UDMH has triggered increasingly severe environmental challenges, highlighting an urgent need for effective treatment measures [12,16,17,18,19].

Prior to developing effective and environmentally friendly remediation strategies, it is imperative to thoroughly characterize the environmental behavior of UDMH, involving adsorption and retention behaviors, as well as its environmental fate and toxicity in contaminated soils. This process-oriented understanding facilitates the design of evidence-based remediation strategies, thereby minimizing the exposure risks associated with UDMH and its transformation metabolites in soil matrices.

Recently, several researchers have completed investigations into soil contamination by UDMH. Kenessov et al. [20] examined the distribution of UDMH transformation products within the sandy soils of fall sites located in Central Kazakhstan (Baikonur Cosmodrome). Their findings revealed that soil contamination was exclusively identified at the epicenters of these fall sites, spanning a diameter of 8–10 m. The surface layer was predominantly characterized by semi-volatile transformation products, ascribable to the rapid evaporation and biodegradation of volatile counterparts. Rodin et al. [21] found that the initial concentration of UDMH in wet soil is higher than that in dry soil. The total UDMH concentration in soils was lower than 0.5% of its initial content regardless of humidity after 90 days from spillage. The research by Krechetov et al. [22] presented that UDMH behavior in soils depended on its properties and concentration and the soil’s composition and features, such as particle size distribution, acid–base properties, clay fraction mineralogy, and organic matter content. Koroleva et al. [23] measured the impact of launch vehicles “Proton” and “Soyuz” from Baikonur Cosmodrome in 2014–2016, revealing that the UDMH concentration was up to 1.5 mg∙kg^−1^ in sporadic soil samples; in the leakage place, the pH value of the soil increased from 6.6 to 8.3–9.4. Researchers [24,25] investigated the transformation products of UDMH when in contact with oxygen in the air. They found that the majority of UDMH would undergo fast oxidative transformation predominantly by the radical mechanism, forming a wide range of related products, which include N-nitrosodimethylamine (NDMA), N, N-dimethylformamide (DMF), dimethylamine (DMA), formaldehyde dimethylhydrazone (FDMH), formic acid dimethylhydrazine (FADMH), and other hazardous substituted heterocyclic compounds. Ul’yanovskii et al. [17,18] investigated the migration of UDMH in histosols of rocket stage fall places in the Russian north (Plesetsk Cosmodrome). The highest concentration of UDMH was detected near the crater’s center, reaching up to 240 mg∙kg^−1^; UDMH and its primary transformation products were strongly bound with natural organic and humic substances, leading to the prolonged persistence of pollution levels. Although the distribution of UDMH and its main transformation products in several fall sites (generally in Kazakhstan or Russia) was the focus of much research, the occurrence behavior of UDMH across various typical soil matrices, the holistic impact of soil physiochemical properties on the adsorption mechanism, the environmental fate of UDMH, and the phytotoxicity remain unclear. The lack of basic data on the adsorption and retention of UDMH in diverse soils and the environmental fate and toxicity of UDMH limits the development of effective application strategies and environmental safety risk assessments.

Therefore, in this study, we aimed to (1) explore the adsorption properties of UDMH across contrasting soil matrices; (2) discuss the relationship between the adsorption behavior of UDMH in soils and the physicochemical characteristics of soil; and (3) evaluate the environmental fate and toxicity of UDMH by environmental toxicity simulation calculation and the germination assay. These results could enhance our knowledge of the environmental fate of UDMH and facilitate field application and environmental risk assessments.

## 2. Materials and Methods

### 2.1. Soil Sample Collection and Physicochemical Characteristics

Soil samples of yellow-brown soil (YS), red soil (RS), and black soil (BS), which can be classified as Luvisols, Ferralsols, and Phaeozems, respectively, according to the international classification (WRB), were collected from northwest (Xi’an, Shanxi), southern (Changsha, Hunan), and northeast (Suihua, Helongjiang) China, respectively. The soil sampling depth ranged from 0 to 25 cm. Before sampling, plant residues on the soil surface and the floating soil in the upper layer were removed. The samples collected from the same region were thoroughly mixed. After removing the large sand particles, gravel, and other debris, the soil samples were air-dried in the shade, passed through a 40-mesh sieve, and then stored for further use. The tests confirmed that no unsymmetrical dimethylhydrazine was detected in the soil samples. The physicochemical properties of the soil samples are presented in Table 1. The soil pH value was measured in the soil/water suspension (1:2, *w*/*v*) using a pH meter (Seven Compact, Mettler Toledo, Greifensee, Switzerland) [26]. The electrical conductivity of the soil was measured by the Electrode Method. The cation exchange capacity of the soil was measured by the method of EDTA-acetate ammonium salt exchange. The soil organic matter was determined by a fully automatic organic matter analyzer (JX-S7066, Jingxin, Shanghai, China). The total contents of phosphorus and potassium, and the contents of available phosphorus and potassium, were measured according to the Methods of Soil Analysis [27,28] by Agilent Technologies 5110 ICP-OES (Agilent, Santa Clara, CA, USA). The contents of ammonia nitrogen and nitrate nitrogen were determined using a UV spectrophotometer (TU-1900, Puxi, Beijing, China), and the total nitrogen was determined using a Fully Automatic Kjeldahl Nitrogen Analyzer (K1160, Hanon, Jinan, China) [28]. The soil particle size was measured using a particle size analyzer and classified according to the USDA particle size grading system, referring to clay (<0.002 mm), silt (0.02–0.002 mm, 0.2–0.02 mm), and sand (2.0–0.2 mm).

The UDMH chemical used in this study was of analytical grade; ultrapure water (18.25 MΩ·cm) was used for the preparation of the solutions. UDMH was determined according to the Water quality–Determination of asymmetrical dimethyl hydrazine–Amino ferrocyanide sodium spectrophotometric method [29].

### 2.2. Methods

#### 2.2.1. Adsorption Kinetics Experiment

The soil adsorption experiments were performed in accordance with a batch equilibrium method by the Organization for Economic Co-operation and Development (OECD) [30]. First, 5 g of yellow-brown soil, red soil, and black soil were separately put into 50 mL centrifuge tubes, and then 20 mL of 200 mg∙L^−1^ UDMH solution was added to each tube to prepare suspension solutions. The samples were shaken at a constant temperature of 25 °C in the absence of light for durations of 1, 2, 4, 8, 12, 24, 36, 48, 56, 60, and 72 h. Afterward, the samples were centrifuged at 5000 rpm for 10 min. The supernatant was filtered through a 0.45 μm filter membrane, and the concentration of UDMH in the filtrate was determined. The pre- and post-adsorption concentrations of UDMH were calculated. Three parallel samples were taken at each sampling time. Blank samples (UDMH solutions without soil) and control samples (soil samples without UDMH) were prepared for the experiment. The soil adsorption amount *Q*_t_ (mg∙kg^−1^) for UDMH at time t was calculated by the following formula:

(1) $$Q_{t}=\frac{V\left(C_{0}-C_{t}\right)}{m}$$

where *V* (mL) denotes the solution volume, *C*_0_ (mg∙L^−1^) represents the initial concentration of UDMH, *C*_t_ (mg∙L^−1^) represents the concentration of UDMH at time t, and *m* (g) indicates the soil mass.

#### 2.2.2. Isothermal Adsorption Experiment

First, 5 g of yellow-brown soil, red soil, and black soil were put into 50 mL centrifuge tubes. Then, different types of soil were individually mixed with 20 mL of UDMH solution at initial concentrations of 25, 50, 100, 150, 200, and 250 mg∙L^−1^. Based on the results of the adsorption kinetics experiment, the samples were agitated until an adsorption equilibrium was reached at a constant temperature of 25 °C in the dark. Then, the samples were centrifuged at 5000 rpm for 10 min, and the supernatant was filtered through a 0.45 μm filter membrane to determine the concentration of UDMH in the filtrate. The pre- and post-adsorption concentrations of UDMH were calculated. Three replicate samples were prepared for each concentration level across all soil types. Blank samples (UDMH solutions without soil) and control samples (soil samples without UDMH) were used for the experiment. The results showed that the losses caused by sample bottle adsorption and solution evaporation could be ignored. The soil adsorption amount *Q*_e_ (mg kg^−1^) for UDMH at the adsorption equilibrium was calculated by the following formula:

(2) $$Q_{e}=\frac{V\left(C_{0}-C_{e}\right)}{m}$$

where *C*_e_ (mg∙L^−1^) denotes the adsorption equilibrium concentration.

#### 2.2.3. Phytotoxicity Assessment

The germination index (GI), as a fast and reliable assay, is used to detect the negative effects of contaminants on plant growth [31,32]. Chinese cabbage (*Brassica rapa* ssp. *chinensis*) seeds of uniform size and plump appearance were selected, and soaked in a hypochlorous acid sodium solution of 5% concentration for 4 to 5 min, rinsed several times with tap water, and dried for the germination assay. Ten treatment groups were established with a UDMH concentration ranging from 0 to 600 mg·L^−1^. Petri dishes (diameter = 100 mm) containing uniformly spread filter paper served as the germination bed. Twenty cabbage seeds were evenly distributed in the germination bed. Three replicate samples were prepared for each treatment; precisely 10 mL of UDMH solution at distinct concentration levels was introduced into each Petri dish. The seeds were subjected to incubation under a 12 h light/12 h dark cycle at 25 °C ± 0.5 °C, with a light intensity of 2000 lx. During the incubation period, the samples were replenished with water in a timely manner, and moldy seeds were promptly removed to ensure adequate solution levels and clean filter papers. The germination status was monitored and recorded.

The germination rates with respect to the control (distilled water) treatment and root lengths were determined after 72 h of incubation. The germination index (GI) was calculated by Equation (3).

*GI* = (*N*_u_ × *L*_u_)/(*N*_c_ × *L*_c_) (3)

where *N*_u_ is the number of germinated seeds in each UDMH sample, *N*_c_ is the number of germinated seeds in the control, *L*_u_ is the mean root length of the germinated seeds in each UDMH sample, and *L*_c_ is the mean root length of the control (CK).

Additionally, germination potential is also an important indicator in the seed germination assay. Germination potential refers to the proportion of seeds that exhibit normal germination relative to the total number of seeds evaluated during the initial phase of the germination assay. Under the stress of UDMH, the number of germinated seeds reached its peak on the second day. Germination potential (*GP*) can be calculated by Equation (4).

*GP* = *N*_u_/*N*_0_ × 100% (4)

where *N*_u_ is the number of germinated seeds in each UDMH sample and *N*_0_ is the number of supplied seeds.

### 2.3. Model Calculation Method and Statistical Analysis

#### 2.3.1. Adsorption Kinetics Calculation

(1) The adsorption kinetics behavior of UDMH in soil is fitted using adsorption kinetics models. The pseudo-first-order model (PFOM) and the pseudo-second-order model (PSOM) are widely used in the study of adsorption kinetics. It is generally accepted that the adsorption process fitting the PFOM is more likely to be a physical adsorption process. The model is applicable to scenarios featuring high initial adsorbate concentrations, the initial stage of adsorption, and adsorption materials with limited active sites. The adsorption process fitting the PSOM is more likely to be a chemical adsorption process. The model is applicable to situations with low initial concentration of adsorbate, the later stage of the adsorption process, and the presence of a large number of active sites in the adsorption material.

The pseudo-first-order kinetics model is expressed as follows:

(5) $$\frac{dQ_{t}}{dt}=k_{1}(Q_{e}-Q_{t})$$

The pseudo-second-order kinetic model is expressed as follows:

(6) $$\frac{dQ_{t}}{dt}=k_{2}{\left(Q_{e}-Q_{t}\right)}^{2}$$

where *Q*_e_ represents the adsorbed amount for UDMH at the adsorption equilibrium, mg·g^−1^; *Q*_t_ represents the adsorbed amount for UDMH at time t, mg·g^−1^; *k*_1_ represents the adsorption rate constant for the pseudo-first-order kinetic model, h^−1^; and *k*_2_ represents the adsorption rate constant for the pseudo-second-order kinetic model, g·mg^−1^·h^−1^.

(2) The isothermal adsorption curve presents the performance of an adsorbent under dynamic equilibrium conditions at a constant temperature. The linear model represents a linear relationship between the equilibrium adsorption concentration and adsorption amount, while the Langmuir model and the Freundlich model are most widely applied as dual-parameter nonlinear adsorption models.

The linear model is expressed as follows:

(7) $$Q_{e}=K_{d}C_{e}$$

The Langmuir model is expressed as follows:

(8) $$\frac{1}{Q_{e}}=\frac{1}{Q_{m}}+\frac{1}{K_{L}Q_{m}C_{e}}$$

The Freundlich model is expressed as follows:

(9) $$lgQ_{e}=1/nlgC_{e}+lgK_{F}$$

where *Q*_e_ represents the adsorbed amount of UDMH at the adsorption equilibrium, mg·kg^−1^; *Q*_m_ denotes the maximal adsorbed amount of DMH by the soil, mg·kg^−1^; *C*_e_ is the concentration of UDMH in the solution at the adsorption equilibrium, mg·L^−1^; *K*_d_ is the constant of adsorption affinity; *K*_L_ is the Langmuir constant; *K*_F_ is the constant of Freundlich adsorption equilibrium; and 1/*n* is a dimensionless parameter (typically ranging from 0 to 1) that characterizes adsorption intensity, where a smaller 1/*n* value indicates better adsorption performance. *K*_F_ and 1/*n* can be derived from the intercept and slope of the Freundlich model, respectively.

#### 2.3.2. Environmental Toxicity Simulation Calculation Method

QSAR (Quantitative Structure–Activity Relationship) modeling was used to predict and assess the sustainable environmental pollution potential and toxicity of the degradation intermediates of UDMH in this study. All computational analyses were performed using EPI Suite V4.11 software, jointly developed by the U.S. Environmental Protection Agency (EPA) (Washington, DC, USA) and SRC, Inc. (Syracuse, NY, USA). The software can be accessed at https://www.epa.gov/tsca-screening-tools/download-epi-suitetm-estimation-program-interface-v411 (accessed on 3 March 2025). In particular, 13 independent modules for estimating organic compound properties and 1 shell software were integrated into this software, enabling the assessment of the environmental persistence and toxicity of chemicals (EPA 2012).

#### 2.3.3. Statistical Analysis

All analyses were conducted in triplicate. Mean values were presented with standard errors. Statistical analysis was processed using SPSS 23.0 software. All figures were drawn by Origin Pro software (version 2024).

## 3. Results and Discussion

### 3.1. Characteristics and Mechanisms of Adsorption for UDMH

Soil is the potential reservoir for UDMH; the environmental fate of UDMH determines its persistence, potential mobility, and bioavailability in soil matrices. Therefore, investigating the adsorption behavior and retention mechanisms of UDMH across different soil matrices is critical for predicting its environmental fate. Model fitting of adsorption kinetics and thermodynamics results for UDMH adsorption by soils enables a systematic investigation of its adsorption behavior.

#### 3.1.1. Adsorption Kinetics

**Figure 1:**
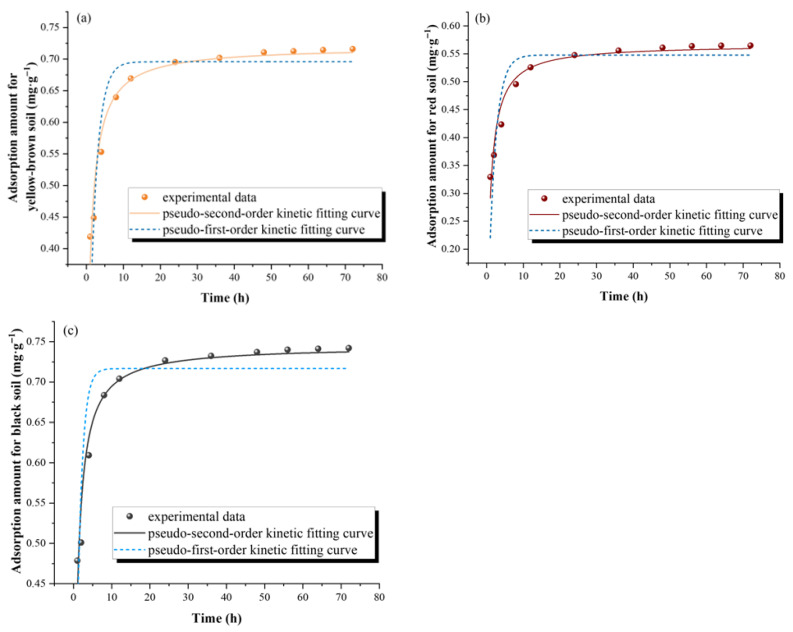
Adsorption kinetics fitting curves. (**a**) is yellow-brown soil, (**b**) is red soil, and (**c**) is black soil.

Adsorption kinetics is often used to present the temporal variation in the pollutant adsorption rate, thereby exploring the adsorption mechanism and potential adsorption rate-limiting steps. The adsorption process of UDMH by soil can be divided into three stages: rapid adsorption, slow adsorption, and adsorption equilibrium (Figure 1a–c). The results showed that the adsorption amount of UDMH in the three soil matrices increased rapidly in the first 8 h, and the adsorption amount reached 90% of the equilibrium adsorption amount at 8 h. After that, the adsorption of UDMH gradually slowed down, and the adsorption amount gradually saturated and remained in a relatively stable state after 36 h. The reaction reached the adsorption equilibrium stage, so the adsorption equilibrium time was determined to be 36 h. During the rapid adsorption stage, the adsorption of UDMH by soil primarily involves two fundamental mechanisms. Firstly, UDMH is distributed between the soil organic matter and the aqueous phase; this partitioning is governed by the thermodynamic equilibrium and the relative affinities of UDMH for organic matter and water. Secondly, physical adsorption by clay minerals constitutes another crucial mechanism. This adsorption arises from the intermolecular forces, particularly van der Waals forces, between UDMH molecules and the surface of clay minerals. It represents a surface phenomenon where UDMH molecules are held onto the clay mineral surface through these weak but significant forces. During the early phase of adsorption, the required activation energy was low, the contaminants were easily absorbed by the soil, the available adsorption sites of the soil surface were sufficient, and the initial concentration of UDMH was high; thus, the adsorption rate was fast. As the adsorption proceeded, the adsorption sites on the soil surface were occupied by a large number of substances, gradually approaching saturation, and the pollutants may have diffused into the internal gaps and micropores of the soil particles. The activation energy required for adsorption was larger compared to the physical adsorption [33,34,35]. Additionally, since UDMH is water-soluble, the presence of water decreased the likelihood of its contact with soil particles and made it harder for it to penetrate into the internal pores of these particles, thereby leading to a rapid reduction in the adsorption rate. When UDMH occupied all available adsorption sites within the soil, leading to adsorption saturation, the system was in a state of dynamic equilibrium. Once the adsorption process attained equilibrium, the adsorption amount remained relatively constant and did not exhibit significant variation with the passage of time.

The equilibrium adsorption amount of UDMH varied in the three soil matrices. Taking the adsorption at an initial concentration of 200 mg∙L^−1^ as an example, the equilibrium adsorption amounts of UDMH in the yellow-brown soil, red soil, and black soil were 0.650 mg∙g^−1^, 0.544 mg∙g^−1^, and 0.704 mg∙g^−1^, respectively. The adsorption amount of UDMH in the black soil was significantly higher than that in the yellow-brown soil and red soil. By characterizing some physical and chemical properties of the soil, it can be inferred that the higher organic matter content in black soil than that in yellow-brown soil and red soil leads to a stronger adsorption capacity of black soil. With a higher organic matter content, there were more available adsorption sites for UDMH, and thus, the adsorption capacity of UDMH by the black soil was stronger. To further elucidate the adsorption process of UDMH in the three soil matrices, kinetic data depicting the adsorption amount variation over time were fitted using pseudo-first-order and pseudo-second-order models. The fitting results are shown in Figure 1a–c, and the relevant fitting parameters are presented in Table 2. The model comparison revealed that pseudo-second-order kinetics better described UDMH adsorption in all the soils, with *R*^2^ values greater than 0.95 and greater than the pseudo-first-order kinetic equation (*R*^2^ = 0.842 for the yellow-brown soil, *R*^2^ = 0.841 for the red soil, and *R*^2^ = 0.774 for the black soil), indicating that the adsorption of UDMH in the soils was governed by a synergistic interplay of multiple mechanisms rather than a single dominant process [16,36,37]. The adsorption of UDMH by the soils involved both physicochemical diffusion and chemisorption processes. The adsorption kinetic constants *k*_2_ of the black soil and red soil samples were significantly higher than those of the yellow-brown soil samples, indicating a faster reaction rate in UDMH adsorption by black soil and red soil. In addition, for organic pollutants including UDMH, an inverse relationship typically exists between adsorption capacity and desorption hysteresis across soil types, as exemplified by the observation that black soils exhibit higher adsorption rates but lower desorption rates compared to yellow-brown soils and red soils. The adsorption characteristics of UDMH by the soils were directly related to the physical and chemical properties of the soils. The significant differences in the physical and chemical properties and organic matter composition of the three soil matrices in this study will inevitably lead to differences in the adsorption process. A further discussion will be presented in subsequent sections.

#### 3.1.2. Adsorption Thermodynamic Characteristics

**Figure 2:**
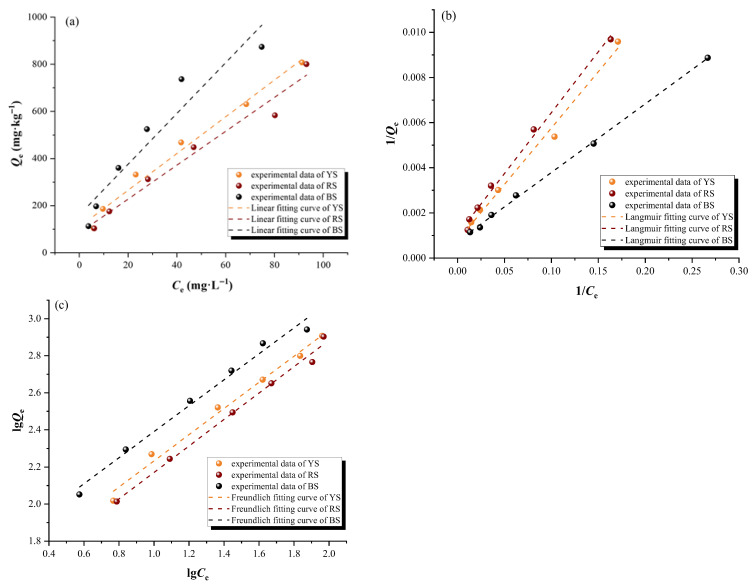
Isothermal adsorption fitting curves. (**a**) is the linear model, (**b**) is the Langmuir model, and (**c**) is the Freundlich model. NOTE: YS, RS, and BS represent yellow-brown soil, red soil, and black soil, respectively.

To further explore the adsorption mechanisms of UDMH in the different soil matrices, isothermal adsorption models, such as the linear, Langmuir, and Freundlich models, were employed to fit the isothermal adsorption process of UDMH. The fitting results are presented in Figure 2a–c, and the relevant fitting parameters are listed in Table 3. The linear model assumes that all adsorption sites on the adsorbent surface have identical energy levels, and the adsorbate molecules exhibit uniform binding affinity to any of these sites. The amount of adsorption shows a linear relationship with the equilibrium concentration. The Langmuir isotherm adsorption model assumes that the adsorption behavior of the adsorbate on the adsorbent surface follows monolayer adsorption, whereas the Freundlich isotherm model describes that the adsorption occurs on heterogeneous surfaces with non-uniform binding sites [36,38,39]. With the increase in UDMH concentrations, the soil adsorption amount exhibited a continuous rise. The adsorption processes of UDMH in all three soil matrices demonstrated a better fit to both the Freundlich and Langmuir isotherm models compared with the linear model. The correlation coefficients (*R*^2^) for the Langmuir isothermal adsorption model ranged from 0.991 to 0.998, *R*^2^ for the Freundlich isothermal adsorption model ranged from 0.986 to 0.992, and those for the linear model ranged from 0.894 to 0.979. These findings indicated that the adsorption of UDMH in the soils involved both monomolecular layer adsorption and multimolecular layer chemical adsorption on heterogeneous surfaces.

Based on the Langmuir isothermal adsorption model, the maximal adsorption amounts of UDMH for the yellow-brown soil, red soil, and black soil were 1.259 mg·g^−1^, 1.000 mg·g^−1^, and 1.428 mg·g^−1^, respectively. It was evident that the black soil exhibited the highest adsorption amount, followed by the yellow-brown soil and red soil. The nonlinear index (*n*) and adsorption coefficient (*K*_F_) values in the Freundlich isothermal adsorption model can be used to describe the adsorption capacity of soils for UDMH [16,34,35]. When *n* < 1, it indicates low adsorption affinity between soil particles and UDMH molecules, suggesting an unfavorable adsorption process. When *n* approaches 1, the adsorption process exhibits linearity. Conversely, when *n* > 1, it indicates high adsorption affinity between soil particles and UDMH molecules, facilitating the adsorption process [33]. According to Table 3, the values of the nonlinear index (*n*) for UDMH adsorption on the three soil matrices were all greater than 1, indicating that the adsorption process of UDMH in the soils was multimolecular layer adsorption on heterogeneous surfaces. For the three soil samples analyzed via the Freundlich model, the values of 1/*n* ranged from 0.700 to 0.713, all less than 1, exhibiting “L-type” isotherm adsorption characteristics. This indicated the strong adsorption affinity between UDMH and soil particles at low concentrations, but as the concentration increased, the adsorption sites were gradually occupied, leading to a gradual decrease in adsorption affinity. Meanwhile, the values of *K*_F_ for UDMH adsorption in the yellow-brown soil, red soil, and black soil were 33.729, 28.721, and 48.955, respectively, indicating that the adsorption capacities of the different soil matrices for UDMH varied, ascribable to the differences in their physicochemical properties. The black soil had the highest *K*_F_ value, suggesting the weaker mobility of UDMH, and it was more likely to remain in the black soil, causing severe pollution. The adsorption affinity constant (*K*_d_) value in the linear model is commonly used to assess the adsorption capacity of soils and to describe the migration ability and bioavailability of contaminants in soils. A higher *K*_d_ value indicates a greater capacity of soil matrices to adsorb contaminants. According to Table 3, the *K*_d_ values for UDMH adsorption in the yellow-brown soil, red soil, and black soil were 7.792, 7.191, and 10.771 L·kg^−1^, respectively. The significantly higher *K*_d_ value for the black soil compared to the yellow-brown soil and red soil further demonstrated its greater adsorption affinity for UDMH.

Integrating kinetic and thermodynamic analyses, the characteristic parameters, such as the adsorption reaction rate, adsorption strength, and adsorption amount, consistently demonstrate a sequential enhancement in UDMH adsorption performance across the soil types: red soil < yellow-brown soil < black soil.

### 3.2. Correlation Analysis of Soil Characteristics and UDMH Adsorption Behavior

**Figure 3:**
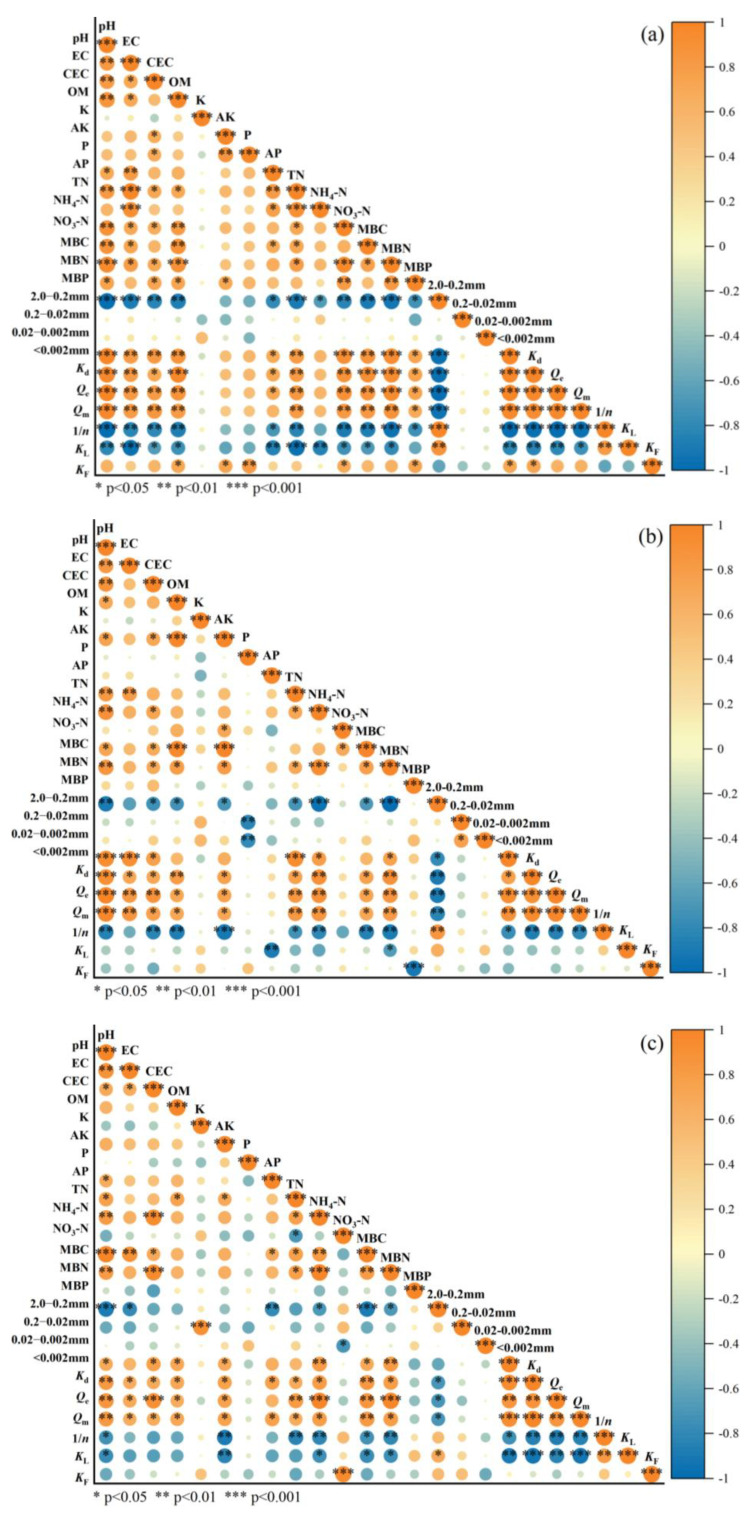
Heatmaps of Spearman correlation coefficients between the physicochemical and adsorption parameters for yellow-brown soil (**a**), red soil (**b**), and black soil (**c**). NOTE: pH, EC, CEC, OM, K, AK, P, AP, TN, NH_4_-N, NO_3_-N, MBC, MBN, and MBP represent pH value, electrical conductivity, cation exchange capacity, organic matter, total potassium, available potassium, total phosphorus, available phosphorus, total nitrogen, ammonium nitrogen, nitrate nitrogen, microbial biomass carbon, microbial biomass nitrogen, and microbial biomass phosphorus, respectively; 2.0–0.2 mm, 0.2–0.02 mm, 0.02–0.002 mm, <0.002 mm represent soil particle size distribution; and *K*_d_, *Q*_e_, *Q*_m_, 1/*n*, *K*_L_, and *K*_F_ represent adsorption affinity constant, equilibrium adsorption amount, maximal adsorption amount, nonlinear index, adsorption constant in the Langmuir isotherm model, and adsorption constant in the Freundlich isotherm model, respectively.

Soil physicochemical properties significantly influence the adsorption, migration, transformation, and biological effects of pollutants in soils [13,22]. The results of the soil physicochemical properties of the yellow-brown soil, red soil, and black soil samples are shown in Table 1. The differences in the adsorption behaviors of UDMH among the various soil types were primarily governed by their distinct physicochemical characteristics. Thus, the Spearman correlation analysis (generally for non-normal data distribution) was used to interpret the relationship between the physicochemical and adsorption parameters for the yellow-brown soil, red soil, and black soil, as illustrated in Figure 3a–c, including pH value, electrical conductivity (EC), cation exchange capacity (CEC), organic matter (OM), potassium (K), available potassium (AK), phosphorus (P), available phosphorus (AP), total nitrogen (TN), ammonium nitrogen (NH_4_-N), nitrate nitrogen (NO_3_-N), microbial biomass carbon (MBC), microbial biomass nitrogen (MBN), and microbial biomass phosphorus (MBP), as well as the soil particle size distribution and the adsorption characteristic parameters, such as equilibrium adsorption amount (*Q*_e_), maximal adsorption amount (*Q*_m_), adsorption affinity constant (*K*_d_), adsorption constant in the Langmuir isotherm model (*K*_L_), adsorption constant in the Freundlich isotherm model (*K*_F_), and the nonlinear index (1/*n*). According to Table 1, the yellow-brown soil (YS) collected from northwest China was alkaline (pH = 7.92), the red soil (RS) from southern China was acidic (pH = 5.20), and the black soil (BS) from northeast China was weakly alkaline (pH = 7.45). This variation stemmed from the distinct geological backgrounds and regional environmental conditions of each soil sample. Soil acidity–alkalinity (pH) could significantly influence the occurrence and transformation of pollutants within the soil environment. A comparison of UDMH adsorption amounts among the three kinds of soil samples presented in Section 3.1.1 revealed that alkaline soils exhibited significantly enhanced retention of UDMH. The electrical conductivity (EC) value of the soil samples represented a combined effect of the ionic concentration and ionic migration rate within the soil matrix, which subsequently influenced the migration behavior of contaminants in the soil environment. There was a highly significant positive correlation between the EC value and the adsorption affinity constant (*K*_d_), maximal adsorption amount (*Q*_m_), and equilibrium adsorption amount (*Q*_e_) (*p* < 0.01), as well as a significant negative correlation with the adsorption constant (*K*_L_) and nonlinear index (1/*n*) (*p* < 0.05) for the yellow-brown soil matrix, as shown in Figure 3a. The nonlinear index (1/*n*) characterizes the adsorption intensity; a smaller 1/*n* value indicates better adsorption performance. This means the variation trends in the 1/*n* value and *K*_d_, *Q*_m_, and *Q*_e_ are generally opposite, as is the adsorption constant (*K*_L_) according to the present adsorption experiment results. There was a highly significant positive correlation between the EC value and the maximal adsorption amount (*Q*_m_) and equilibrium adsorption amount (*Q*_e_) (*p* < 0.01) and a significant positive correlation with the adsorption affinity constant (*K*_d_) (*p* < 0.05) for the red soil matrix, as shown in Figure 3b. Similarly, there was a significant positive correlation between the EC value and *Q*_m_, *Q*_e_, and *K*_d_ (*p* < 0.05) for the black soil matrix, as shown in Figure 3c. According to Table 1, the EC values followed the trend of BS (33.8 mS m^−1^) > YS (17.7 mS m^−1^) > RS (5.7 mS m^−1^), indicating that UDMH tended to remain in the soils with high ion concentrations and ion migration rates. The cation exchange capacity (CEC) of soil enabled it to regulate soil solution pH and buffer against external acid–base fluctuations. Meanwhile, the CEC value served as a critical indicator for evaluating soil fertility retention and buffering capacity, reflecting the soil’s ability to adsorb and store cations (e.g., potassium, calcium, magnesium, ammonium). A higher CEC value indicates greater soil capacity to adsorb and retain cationic nutrients, enhancing fertility preservation. Table 1 shows that the CEC value of the BS (233.6 mmol·kg^−1^) was significantly higher than that of the YS (164.0 mmol·kg^−1^) and RS (186.4 mmol·kg^−1^). The BS also had the highest nutrient contents of K, AK, P, AP, TN, NH_4_-N, and NO_3_-N, followed by the YS and then the RS. The nutrient composition of soil is not only correlated with its cation exchange capacity (CEC) but also significantly influenced by disparities arising from ecological environments, geological conditions, and anthropogenic agricultural practices. Figure 3a reveals a highly significant positive correlation between the soil CEC and the equilibrium adsorption amount (*Q*_e_) and maximal adsorption amount (*Q*_m_) (*p* < 0.01), as well as a significant positive correlation with the AK, P, TN, MBN, and MBP contents and the adsorption affinity constant (*K*_d_) (*p* < 0.05) and a significant negative correlation was presented with 1/*n* and *K*_L_ (*p* < 0.05) for the yellow-brown soil matrix. Figure 3b presents a highly significant positive correlation between the soil CEC and the equilibrium adsorption amount (*Q*_e_) (*p* < 0.01), as well as a significant positive correlation with the AK, TN, NH_4_-N, MBN, and MBC contents, the adsorption affinity constant (*K*_d_), and the maximal adsorption amount (*Q*_m_) (*p* < 0.05); a highly significant negative correlation is also presented with 1/*n* (*p* < 0.01) for the red soil matrix. Figure 3c shows an extremely significant positive correlation between the soil CEC, the equilibrium adsorption amount (*Q*_e_), and the NH_4_-N and MBN contents (*p* < 0.001), as well as a significant positive correlation with the MBC content, the adsorption affinity constant (*K*_d_), and the maximal adsorption amount (*Q*_m_) (*p* < 0.05). Additionally, microbial biomass carbon (MBC) was an important component of the soil organic carbon pool, microbial biomass nitrogen (MBN) was a crucial reserve of soil nitrogen, and microbial biomass phosphorus (MBP) was a significant source of available phosphorus in the soils. The contents of MBC, MBN, and MBP in the soil samples were important indicators of soil fertility, participating in soil nutrient transformation and cycling while directly influencing soil physicochemical properties and the migration and transformation of soil contaminants. According to Table 1, the contents of MBC, MBN, and MBP in the soil samples also followed the trend of BS (110 mg·kg^−1^) > YS (57.1 mg·kg^−1^) > RS (17.3 mg·kg^−1^) for MBC, BS (92.0 mg·kg^−1^) > YS (22.2 mg·kg^−1^) > RS (11.9 mg·kg^−1^) for MBN, and BS (0.782 mg·kg^−1^) > YS (0.646 mg·kg^−1^) > RS (0.507 mg·kg^−1^) for MBP. Figure 3a,c indicates a highly significant positive correlation between the soil MBC content and *K*_d_, *Q*_m_, and *Q*_e_ (*p* < 0.01), as well as a significant negative correlation with *K*_F_ and 1/*n* (*p* < 0.05) for the yellow-brown and black soil matrices. Figure 3b shows a significant positive correlation between the soil MBC content and *K*_d_, *Q*_m_, and *Q*_e_ (*p* < 0.05), as well as a highly significant negative correlation with 1/*n* (*p* < 0.01). In addition, a highly significant positive correlation was found between the soil MBN content and *K*_d_, *Q*_m_, and *Q*_e_ (*p* < 0.05) for the red soil matrix. An extremely significant positive correlation was shown between the soil MBN content and *Q*_e_ (*p* < 0.001), as well as a significant positive correlation between the MBN content and *K*_d_ and *Q*_m_, and a highly significant negative correlation with 1/*n* (*p* < 0.01) for the black soil matrix. These findings were consistent with the high adsorption affinity, adsorption strength, and adsorption amount of UDMH onto the BS matrix. Soil organic matter (SOM) is an important nutrient source for plants, containing various essential elements, such as carbon, nitrogen, phosphorus, and sulfur, required for plant growth. As organic matter decomposes, these nutrients are gradually released, providing a continuous nutrient supply for plants, particularly playing a key role in the cycling of nitrogen and phosphorus. SOM exhibits strong hydrophilicity, enabling it to absorb and retain substantial moisture volumes, thereby significantly enhancing the soil’s water-holding capacity. According to Table 1, the SOM contents of the three soils followed the trend of BS (24.1 g·kg^−1^) > YS (22.0 g·kg^−1^) > RS (8.86 g·kg^−1^); the SOM contents of the BS and YS were similar, but the RS had a significantly lower SOM content. Figure 3a shows an extremely significant positive correlation between the SOM content and *K*_d_ (*p* < 0.001), a highly significant positive correlation with *Q*_e_ and *Q*_m_ (*p* < 0.01), and a significant negative correlation with *K*_L_ and 1/*n* (*p* < 0.05) for the yellow-brown soil matrix. Figure 3b presents a highly significant positive correlation between the SOM content and *K*_d_ (*p* < 0.01), a significant positive correlation with *Q*_e_ and *Q*_m_ (*p* < 0.05), and a highly significant negative correlation with 1/*n* (*p* < 0.01) for the red soil matrix. Figure 3c reveals a significant positive correlation between the SOM content and *K*_d_, *Q*_e_, and *Q*_m_ (*p* < 0.05) for the black soil matrix. Thus, the elevated soil organic matter content in the BS represented a key determinant underlying its superior adsorption capacity and enhanced binding affinity for UDMH. Furthermore, since UDMH is water-soluble and SOM exhibited strong hydrophilicity, this also facilitated UDMH adsorption. Essentially, the influence of SOM on UDMH adsorption behavior was mainly attributed to its deprotonated functional groups (e.g., -COO^−^), which provided potential adsorption sites for UDMH molecules. UDMH can also be adsorbed through hydrogen bonding interactions with polar functional groups of SOM or by adding its electron-rich groups and electron-deficient sites to SOM [39,40,41,42]. Therefore, the soil matrices with higher organic matter content exhibited greater adsorption capacity for UDMH. In addition, particle size analysis of each soil sample (Table 1) revealed that the YS had the largest proportions of particle sizes referring to <0.002 mm (15.68%) and 0.02–0.002 mm (39.14%), and it was clay with fine particles. The RS had relatively large proportions of particle sizes, referring to 2.0–0.2 mm (15.92%) and 0.2–0.02 mm (46.37%), classifying it as sandy soil with larger particles. The particle size distribution trend of the BS was between that of the YS and RS. The soil particle size distribution directly governs the speciation and loading of contaminants on soil surfaces, thereby modulating their transport behavior within the soil matrix through altered interfacial interactions and pore-scale accessibility. As shown in Figure 3a–c, soil particles in the range of 2.0–0.2 mm exhibited a significant negative correlation with *Q*_e_, *Q*_m_, and *K*_d_ (*p* < 0.05) and a significant positive correlation with 1/*n* (*p* < 0.05); inversely, soil particles in the range of <0.002 mm exhibited a significant positive correlation with *Q*_e_, *Q*_m_, and *K*_d_ (*p* < 0.05) and a significant negative correlation with 1/*n* (*p* < 0.05) for the three kinds of soil matrices. Large soil particles generally have a smaller superficial area, which might lead to weak adsorption, but small soil particles have a greater superficial area, which is beneficial for adsorption. It can be suggested that the soil particle distribution influenced the maximal adsorption amount and adsorption strength of UDMH. Comparing the particle size distributions of the three soil matrices, the YS and BS had finer soil particles, facilitating UDMH adsorption, whereas the RS had larger particle sizes, resulting in relatively lower UDMH adsorption amounts. Other studies have shown that a higher clay content in soil implied greater porosity, smaller particle size, larger mineral-specific surface areas, and more adsorption sites, all of which were conducive to increasing the adsorption reaction rate and amount of soil adsorbents [38,43]. This was also an important reason for the high UDMH adsorption capacity of the YS and BS.

### 3.3. Environmental Fate and Toxicity of UDMH

Given the ecological and environmental hazards posed by UDMH and its intermediate metabolites generated during the metabolic process, an assessment of the environmental toxicity effects of UDMH is necessary. Based on the Quantitative Structure–Activity Relationship (QSAR) method, computational toxicology modeling software (EPI Suite V4.11) was employed to evaluate the environmental persistence and environmental toxicity of UDMH and its intermediate metabolites. Meanwhile, the germination index evaluation method was utilized to assess the biological toxicity of UDMH. The seeds were extremely vulnerable during early germination and were highly sensitive to the toxic substances in the environment. As a comprehensive biological indicator, the germination index not only reflected the germination capacity of the seeds but also indirectly indicated the potential biological toxicity in the environment.

#### 3.3.1. Environmental Fate and Toxicity Simulation Calculation

The octanol/water partition coefficient (*K*_ow_) can be estimated using the KOWWIN^TM^ model within EPI Suite V4.11 software. The KOCWIN^TM^ model was employed to predict the soil and sediment adsorption coefficient. BioWin software is capable of biodegradability prediction (EPI Suite V4.11). The sub-model variable of Biodegradation Prediction by Biowin 3 (BDP3) within the ultimate degradation model framework can be used to calculate the probability of pollutant biodegradation, thereby assessing biodegradation rates. A lower BDP3 value indicates a reduced probability of pollutant biodegradation and a longer duration for biodegradation in the environment. The BDP3 value correlates with the time required for pollutant biodegradation, corresponding to distinct half-life classification tiers that quantify environmental persistence, including hours, hours to days, days, days to weeks, weeks, weeks to months, months, and recalcitrant, thus distinguishing the biodegradation susceptibility of pollutants in the environment. Preliminary assessment of compound environmental persistence can be achieved through BDP3 sub-model variables. Pollutants with a biodegradation half-life exceeding 180 days and a relative BDP3 value below 1.75 exhibit a high environmental persistence; compounds with a biodegradation half-life ranging from 60 to 180 days and a relative BDP3 value between 1.75 and 2.25 demonstrate a moderate environmental persistence. However, in order to assess whether a pollutant is readily biodegradable, the MITI model must also be employed for prediction. This model selects 884 kinds of hazardous chemicals by actual experiments (following the OECD standard test method, OECD 301C) and utilizes quantum chemical structure–activity relationships to predict the biodegradability of most compounds. Generally, a calculation result of the MITI model greater than 0.5 indicates that the compound is not readily biodegradable. The Bioconcentration Factor (*BCF*) can be used to calculate bioaccumulation potential, reflecting the amounts of toxic pollutants metabolized and accumulated within organisms. A multi-media model employing a Level III Fugacity Approach can calculate the degradation half-life of pollutants in soil. The toxicity analysis of UDMH and its primary degradation products towards aquatic organisms can be conducted using the ECOSAR program within EPI Suite V4.11. The ECOSAR calculation program operates independently and calculates both the acute and chronic toxicity of pollutants by determining the octanol/water partition coefficient. The assessment of acute and chronic toxicity for pollutants is based on their impact on specific fish species (including both freshwater and marine fish), water fleas, green algae, earthworms, and other organisms.

The prediction of a compound’s physicochemical properties using the Quantitative Structure–Activity Relationship (QSAR) method is based on the physicochemical data of a large number of compounds, summarizing the patterns exhibited by their macroscopic structures. QSAR research can also display the structural factors that play a decisive role at the microscopic level, thereby identifying the key points in the structure–activity relationship. Consequently, preventive and remedial measures can be specifically conducted based on the structural characteristics to effectively control and prevent pollution. Table 4 presents the physicochemical parameters of UDMH and its degradation products, including water solubility (*S*_W_), Henry’s law constant (*H*), vapor pressure (*P*), the octanol/water partition coefficient (*K*_OW_), the organic carbon adsorption coefficient (*K*_OC_), and the bioaccumulation factor (*BCF*). As indicated in Table 4, almost all compounds exhibited great solubility (*S*_W_), low octanol/water partition coefficients (log*K*_OW_), and low Henry’s law constants (*H*). It can be observed that these substances all exhibit relatively high hydrophilicity and strong water solubility. Consequently, their organic carbon–water partition coefficients (*K*_OC_) and bioconcentration factors (*BCF*) in soil or sediment were correspondingly low. This indicated that these compounds were less adsorbed by soil organic carbon and were prone to being dissolved in soil water and migrating. Meanwhile, they were also less likely to bioaccumulate in aquatic organisms. In terms of vapor pressure, except for Dimethylamine, the vapor pressures (*P*s) of the other compounds were not very high, suggesting that these compounds exhibit a low tendency to escape from the aqueous phase. However, soil is special; once these compounds are presented in the surface layer of dry soil, they may cause severe ecological and environmental pollution.

Generally, the environmental persistence of pollutants is closely related to their degradation potential, and the sub-model variable BDP3 (Biowin3) value of the ultimate degradation model, half-life, and biodegradability for pollutants were important influencing factors. Table 5 presents the key indicators calculated using EPI Suite V4.11 software. Apart from formic acid, which has a biodegradation time ranging from days to weeks, and Dimethylaminoacetonitrile and N-nitrosodimethylamine, which exhibit biodegradation times spanning weeks to months, the biodegradation time for most substances falls within the range of weeks. The BioWin program was employed to predict the biodegradability of UDMH and its major degradation products, revealing that UDMH, acetaldehyde dimethylhydrazone, Dimethylaminoacetonitrile, and N-nitrosodimethylamine were not readily biodegradable, significantly increasing their likelihood of persisting in the environment. According to literature reports, considerable concentrations of UDMH and its degradation products were still detected in dry soil 30 years after a propellant leakage incident [44]. It was speculated that when the concentration of UDMH increased to a certain level, it greatly reduced the degradation activity of indigenous microorganisms in the soil. After UDMH was oxidized to form its major degradation products, microorganisms in the soil were insufficient to decompose and mineralize these toxic and harmful substances, allowing these pollutants to persist in the soil for an extended period. By invoking the fugacity algorithm (Level III) module in EPI Suite software and using soil and sediment as media, the distribution and migration trends of various pollutants in soil and sediment were deduced based on the physicochemical parameters of each pollutant through fugacity and mass balance equations. The degradation half-life of UDMH and its major degradation products in soil and sediment can be calculated by the fugacity model program (Fugacity). In the soil medium, except for formic acid, with a degradation half-life of 416 h, and Dimethylaminoacetonitrile and N-nitrosodimethylamine, with half-lives of 1800 h, the half-lives of the other pollutants in the soil were all 720 h. In the sediment medium, except for formic acid, with a degradation half-life of 1870 h, and Dimethylaminoacetonitrile and N-nitrosodimethylamine, with half-lives of 8100 h, the half-lives of the other pollutants in the sediment were all 3240 h. According to the current established criteria for environmental persistence assessment, only half-life was considered. Therefore, Dimethylaminoacetonitrile and N-nitrosodimethylamine can be classified as highly persistent pollutants in the environment (t_1/2_, sediment > 180 days), while the rest can be considered moderately persistent pollutants (t_1/2_, 60 days < sediment < 180 days). In summary, from various perspectives, UDMH, acetaldehyde dimethylhydrazone, Dimethylaminoacetonitrile, and N-nitrosodimethylamine were not readily biodegradable and could significantly impact the ecological environment. However, based on environmental persistence criteria, only Dimethylaminoacetonitrile and N-nitrosodimethylamine were in line with the criteria and were classified as highly persistent environmental pollutants.

The environmental toxicity of UDMH and its major degradation products can be evaluated based on the Bioaccumulation Factor (BCF) calculated by the BCFBAF model in EPI Suite V4.11 and the biotoxicity parameters calculated by the ECOSAR model. The BCF presents the metabolized and accumulated amounts of pollutants in organisms and is closely related to K_OW_. As indicated in Table 4, all pollutants have low K_OW_ values. According to the estimation formula, when the logK_OW_ of a pollutant is less than 1, the corresponding bioaccumulation parameter logBCF equals 0.5. If 3.0 < logBCF < 3.7, the pollutant shows a moderate bioaccumulation trend; if logBCF > 3.7, the pollutant has a significant tendency for bioaccumulation; and if logBCF < 3.0, the pollutant lacks a bioaccumulation tendency [45]. Therefore, it can be inferred that UDMH and its major degradation products do not exhibit bioaccumulation potential. In addition, the ECOSAR environmental toxicity model can provide both acute toxicity benchmark parameters for pollutants and acute toxicity parameters for fish, water fleas, green algae, earthworms, and other organisms. Table 6 presents the acute toxicity, chronic toxicity, and toxicity parameters for earthworms calculated using the ECOSAR environmental toxicity model. Among these, the acute toxicity value LC_50_ (mg∙L^−1^) represents the median lethal concentration, EC_50_ (mg∙L^−1^) represents the median effective concentration, ChV (mg∙L^−1^) represents the chronic toxicity value, and toxicity to earthworms is expressed as LC_50_ (mg·kg^−1^·dw^−1^). According to the literature [46], when the characteristic toxicity concentration of a pollutant is less than 1 mg∙L^−1^, it can be considered environmentally toxic. As shown in Table 6, among UDMH and its series of degradation products, formaldehyde dimethylhydrazone, acetaldehyde dimethylhydrazone, and formaldehyde hydrazone exhibited green algae acute toxicity, with EC_50_ values less than 1 mg/L, indicating strong environmental acute toxicity. Meanwhile, 1,1-Dimethylhydrazine, Tetramethylhydrazine, formaldehyde dimethylhydrazone, acetaldehyde dimethylhydrazone, formaldehyde hydrazone, dimethylamine, and N,N-dimethylformamide demonstrated strong environmental chronic toxicity. Considering the comprehensive toxicity parameters, formaldehyde dimethylhydrazone, acetaldehyde dimethylhydrazone, and formaldehyde hydrazone exhibited both high acute and chronic toxicity, with formaldehyde dimethylhydrazone showing the greatest environmental toxicity and thus warranting particular attention.

#### 3.3.2. Phytotoxicity Evaluation

**Figure 4:**
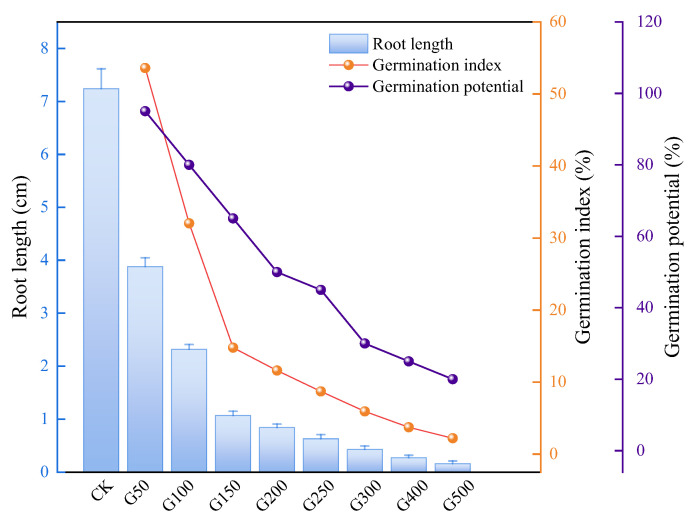
Results of the germination assay. NOTE: CK represents the control sample (no UDMH), G50, G100, G150, G200, G250, G300, G400, and G500 represent the UDMH addition into the germination bed of 50 mg·kg^−1^, 100 mg·kg^−1^, 150 mg·kg^−1^, 200 mg·kg^−1^, 250 mg·kg^−1^, 300 mg·kg^−1^, 400 mg·kg^−1^, and 500 mg·kg^−1^, respectively.

The germination assay and early-stage seedling growth are commonly used to evaluate the phytotoxic impact of contamination on plant growth. According to a reference standard germination index (GI) reported by Emino and Warman [47], GI values over 80% suggest no phytotoxicity, GI values between 50% and 80% suggest moderate phytotoxicity, and GI values below 50% suggest strong phytotoxicity. The root length and germination indexes of the UDMH-contaminated samples were compared with the control sample in this study. As seen in Figure 4, the root length of the control sample of cabbage seeds was 7.42 cm. The root length of the samples exposed to varying amounts of UDMH contamination were 3.88 cm, 2.32 cm, 1.07 cm, 0.84 cm, 0.63 cm, 0.43 cm, 0.27 cm, and 0.16 cm, respectively, for the UDMH addition of 50 mg·kg^−1^ (G50), 100 mg·kg^−1^ (G100), 150 mg·kg^−1^ (G150), 200 mg·kg^−1^ (G200), 250 mg·kg^−1^ (G250), 300 mg·kg^−1^ (G300), 400 mg·kg^−1^ (G400) and 500 mg·kg^−1^ (G500). With 600 mg·kg^−1^ UDMH addition (G600), the seeds had not germinated. The GI values of the samples were 53.59%, 32.04%, 14.78%, 11.60%, 8.70%, 5.94%, 3.73%, 2.21%, and 0%, respectively, corresponding to the increasing UDMH addition of 50 mg·kg^−1^, 100 mg·kg^−1^, 150 mg·kg^−1^, 200 mg·kg^−1^, 250 mg·kg^−1^, 300 mg·kg^−1^, 400 mg·kg^−1^, 500 mg·kg^−1^, and 600 mg·kg^−1^. The GI value of the sample with 50 mg·kg^−1^ UDMH addition was between 50% and 80%, presenting a moderate phytotoxicity, and the GI values of the other high-concentration UDMH-contaminated samples were all below 50%, indicating a strong phytotoxicity of UDMH. Obviously, with increasing UDMH contamination, the GI value sharply declined, indicating that UDMH has a significant impact on plant growth; in particular, when the UDMH addition is over 100 mg·kg^−1^, the phytotoxicity of UDMH will cause serious harm to plants. The germination potential (GP) reflects the ability of seeds to germinate within a short period of time, demonstrating the vitality and germination speed of seeds. Thus, to a certain extent, it reflects the impact of pollutants on the stress of plant growth. It can be seen in Figure 4 that the germination potential (GP) values of the samples obviously decreased with increasing UDMH addition on the second day, during which the seeds germinated most vigorously. When the addition of UDMH was over 200 mg·kg^−1^, the GP values were all below 50%, suggesting a significant impact of UDMH contamination on the initial germination of seed, ascribable to the phytotoxicity of UDMH and the contamination sensitivity of the seed. According to the results of the germination assay, more than 100 mg·kg^−1^ of UDMH in the soil will lead to strong phytotoxicity to plants, and more than 200 mg·kg^−1^ of UDMH will significantly affect the early germination of seeds.

## 4. Conclusions

The investigation of the adsorption behavior of UDMH in typical soil matrices showed that the adsorption performance of red soil, yellow-brown soil, and black soil for UDMH increased sequentially according to the results of the kinetic and thermodynamic studies. In terms of the markedly pronounced correlation between the physicochemical and adsorption parameters for yellow-brown soil, red soil, and black soil, a significant impact of soil physicochemical properties (pH, EC, OM, N, P, K, MBC, particle distribution, and so on) on the adsorption behavior of UDMH in soils was revealed. The simulation calculation of the environmental fate and toxicity of UDMH indicated that UDMH and its transformation products were prone to being dissolved in soil water and migrating; however, the compounds retained in dry soil would lead to severe ecological and environmental contamination. The characteristic difficult biodegradability of UDMH, acetaldehyde dimethylhydrazone, Dimethylaminoacetonitrile, and N-nitrosodimethylamine might result in a conspicuous influence on the ecological environment. Additionally, 1,1-Dimethylhydrazine demonstrated strong environmental chronic toxicity. Formaldehyde dimethylhydrazone, acetaldehyde dimethylhydrazone, and formaldehyde hydrazone exhibited both high acute and chronic toxicity, with formaldehyde dimethylhydrazone showing the greatest environmental toxicity and thus warranting particular attention. The germination assay for phytotoxicity evaluation demonstrated that more than 100 mg·kg^−1^ of UDMH in soil would contribute to strong phytotoxicity, and more than 200 mg·kg^−1^ of UDMH would significantly affect the early germination of seeds. Hence, this research provided helpful insights and theoretical support for the environmental fate and remediation of UDMH.

The obtained results were of great importance for understanding UDMH’s environmental impact. They can serve as a basis for future research aimed at studying the mechanism and kinetics of UDMH binding and transformation in soils of various types in different regions, identifying the specific transformation products, evaluating their toxicities, and exploring soil remediation.

## Figures and Tables

**Table 1:** Physicochemical properties of the soils.

Items	pH	EC (mS m^−1^)	CEC (mmol∙kg^−1^)	OM (g∙kg^−1^)	K (g∙kg^−1^)	AK (mg∙kg^−1^)	P (g∙kg^−1^)
YS	7.93	17.7	164.0	22.0	21.3	258	0.705
RS	5.2	5.7	186.4	8.86	19.3	52	0.379
BS	7.45	33.8	233.6	24.1	22.0	345	0.785
**Items**	**AP** **(mg∙kg^−1^)**	**TN** **(mg∙kg^−1^)**	**NH_4_-N** **(mg∙kg^−1^)**	**NO_3_-N** **(mg∙kg^−1^)**	**MBC** **(mg∙kg^−1^)**	**MBN** **(mg∙kg^−1^)**	**MBP** **(mg∙kg^−1^)**
YS	7.6	1328	3.07	80.9	57.1	22.2	0.646
RS	1.1	938.6	1.59	42.4	17.3	11.9	0.507
BS	72.6	1552	3.91	260	110	92.0	0.782
**Items**	**(2.0~0.2 mm), %**	**(0.2~0.02 mm), %**		**(0.02~0.002 mm), %**		**(<0.002 mm), %**	**Density** **(g∙cm^−3^)**
YS	6.26	38.92		39.14		15.68	2.6
RS	15.92	46.37		36.22		1.49	2.6
BS	15.01	40.20		35.66		9.13	2.6

NOTE: YS, RS, and BS represent yellow-brown soil, red soil, and black soil, respectively. EC, CEC, and OM represent conductivity, cation exchange capacity, and organic matter content, respectively. K, AK, P, and AP represent the content of total potassium, available potassium, total phosphorus, and available phosphorus, respectively. TN, NH_4_-N, and NO_3_-N represent the content of total nitrogen, ammonia nitrogen, and nitrate nitrogen, respectively. MBC, MBN, and MBP represent the content of microbial carbon, nitrogen, and phosphorus, respectively.

**Table 2:** Parameters for the adsorption kinetics model fitting.

Samples	Pseudo-First-Order Kinetic Model			Pseudo-Second-Order Kinetic Model		
	*k*_1_ (h^−1^)	*Q*_e_ (mg·g^−1^)	*R* ^2^	*k*_2_ (g·mg^−1^·h^−1^)	*Q*_e_ (mg·g^−1^)	*R* ^2^
YS	0.511	0.696	0.842	1.459	0.720	0.959
RS	0.514	0.548	0.841	1.868	0.567	0.957
BS	0.781	0.717	0.774	1.858	0.745	0.950

NOTE: YS, RS, and BS represent yellow-brown soil, red soil, and black soil, respectively.

**Table 3:** Parameters for the adsorption thermodynamic model fitting.

Samples	Linear Model		Langmuir Model			Freundlich Model		
	*K*_d_ (L·kg^−1^)	*R* ^2^	*K*_L_ (L·mg^−1^)	*Q*_m_ (mg·g^−1^)	*R* ^2^	*K*_F_	1/*n*	*R* ^2^
YS	7.792	0.979	0.016	1.259	0.991	33.729	0.705	0.989
RS	7.191	0.963	0.020	1.000	0.994	28.721	0.713	0.992
BS	10.771	0.894	0.014	1.428	0.998	48.955	0.700	0.986

NOTE: YS, RS, and BS represent yellow-brown soil, red soil, and black soil, respectively.

**Table 4:** Physicochemical parameters of UDMH and its transformation products.

Compounds	Molecular Formula	CAS No.	*S*_W_ (mg·L^−1^)	*H* (atm·m^3^·mol^−1^)	*P* (kPa)	log*K*_OW_	log*K*_OC_	log*BCF*
1,1-Dimethylhydrazine	C_2_H_8_N_2_	57-14-7	1.0 × 10^6^	4.409 × 10^−5^	21.7	−1.19	1.077	0.5
Tetramethylhydrazine	C_4_N_2_H_12_	6415-12-9	3.467 × 10^5^	4.383 × 10^−5^	17.1	−0.52	1.127	0.5
Formaldehyde dimethylhydrazone	C_3_N_2_H_8_	2035-89-4	3.525 × 10^4^	8.883 × 10^−4^	43.7	0.68	1.358	0.5
Acetaldehyde dimethylhydrazone	C_4_N_2_H_10_	7422-90-4	5.799 × 10^4^	1.569 × 10^−4^	10.3	0.4	1.618	0.5
Formaldehyde hydrazone	CN_2_H_4_	6629-91-0	1.305 × 10^5^	2.026 × 10^−4^	60.5	0.004	1.337	0.5
Formic acid	CH_2_O_2_	64-18-6	1.0 × 10^6^	2.276 × 10^−6^	5.68	−0.46	0	0.5
2-Butanone	C_4_H_8_O	78-93-3	2.11 × 10^5^	1.223 × 10^−4^	12.1	0.256	0.654	0.5
Dimethylaminoacetonitrile	C_4_N_2_H_8_	926-64-7	1.0 × 10^6^	7.881 × 10^−7^	0.875	−0.4338	0.738	0.5
N-Nitrosodimethylamine	C_2_N_2_H_6_O	62-75-9	1.0 × 10^6^	1.116 × 10^−6^	0.36	−0.639	1.358	0.5
Dimethylamine	C_2_NH_7_	124-40-3	1.63 × 10^6^	1.77 × 10^−5^	203	−0.173	0.912	0.5
Formamide	CNH_3_O	75-12-7	1.0 × 10^6^	7.763 × 10^−9^	0.00813	−1.61	0	0.5
N, N-Dimethylformamide	C_3_NH_7_O	68-12-2	1.0 × 10^6^	3.462 × 10^−7^	0.516	−0.934	0	0.5
Dimethylcyanamide	C_3_N_2_H_6_	1467-79-4	1.818 × 10^5^	1.081 × 10^−6^	0.259	−0.1336	0.69	0.5
1,1,4,4-Tetramethyl-2-tetrazene	C_4_N_4_H_12_	6130-87-6	2.647 × 10^4^	1.23 × 10^−4^	2.67	0.694	0.992	0.5

**Table 5:** Environmental persistence of UDMH and its transformation products.

Compounds	Model-Predicted Soil Half-Life (h)	Model-Predicted Sediment Half-Life (h)	BDP3	Model-Predicted Biodegradation Half-Life	Is It Easily Biodegradable?
1,1-Dimethylhydrazine	720	3240	3.0664	weeks	NO
Tetramethylhydrazine	720	3240	3.0044	weeks	YES
Formaldehyde dimethylhydrazone	720	3240	3.0398	weeks	YES
Acetaldehyde dimethylhydrazone	720	3240	3.0088	weeks	NO
Formaldehyde hydrazone	720	3240	3.1018	weeks	YES
Formic acid	416	1870	3.4621	days–weeks	YES
2-Butanone	720	3240	3.0173	weeks	YES
Dimethylaminoacetonitrile	1800	8100	2.6761	weeks–months	NO
N-Nitrosodimethylamine	1800	8100	2.6503	weeks–months	NO
Dimethylamine	720	3240	3.1240	weeks	YES
Formamide	720	3240	3.0454	weeks	YES
N,N-Dimethylformamide	720	3240	2.9834	weeks	YES
Dimethylcyanamide	720	3240	3.0443	weeks	YES
1,1,4,4-Tetramethyl-2-tetrazene	720	3240	2.9425	weeks	YES

**Table 6:** Toxicity parameters of UDMH and its transformation products.

Compounds	LC_50_ (mg∙L^−1^)		EC_50_ (mg∙L^−1^)	ChV (mg∙L^−1^)			LC_50_ (mg∙kg^−1^∙dw^−1^)
	Fish (96 h) Acute ^a^ /Baseline ^b^	Daphnid (48 h) Acute ^a^ /Baseline ^b^	Green Algae (96 h) Acute ^a^ /Baseline ^b^	Fish Chronic ^c^/ Baseline ^b^	Daphnid Chronic ^c^/ Baseline ^b^	Green Algae Chronic ^c^/Baseline ^b^	Earthworm
1,1-Dimethylhydrazine	8.056 ^a^ 36,392.77 ^b^	79.28/15,387.91	1.574/3387.64	1.148/2512.41	9.98/660.62	0.319/460.12	_
Tetramethylhydrazine	6.757/13,160.76	56.37/5923.76	1.411/1688.80	0.788/978.10	6.227/302.65	0.312/263.64	_
Formaldehyde dimethylhydrazone	2.06/908.01	12.83/456.42	0.484/205.40	0.168/76.87	1.126/31.71	0.125/41.0	_
Acetaldehyde dimethylhydrazone	3.103/1939.33	20.70/949.84	0.709/383.98	0.276/159.23	1.917/61.39	0.177/72.35	_
Formaldehyde hydrazone	2.198/2243.61	16.151/1059.55	0.483/368.41	0.220/176.46	1.614/61.87	0.114/64.01	_
Formic acid	6128.83 ^b^	2772.75 ^b^	807.35 ^b^	458.24 ^b^	143.69 ^b^	127.48 ^b^	144.0
2-Butanone	2181.62 ^b^	1054.51 ^b^	403.66 ^b^	176.36 ^b^	65.70 ^b^	73.86 ^b^	190.12
Dimethylaminoacetonitrile	645.62/10,685.8	57.97/4844.57	83.79/1422.93	89.17/800.95	3.615/252.53	22.68/225.74	_
N-Nitrosodimethylamine	770.51/14,257.56	67.58/6345.08	102.31/1726.07	114.28/1045.54	4.12/314.10	27.23/262.74	_
Dimethylamine	231.70/3310.12	21.45/1537.88	29.18/499.81	29.13/255.38	1.379/85.81	0.074/83.73	_
Formamide	5140.35/64,724.62	29,875.42/26,331.54	74.18/4942.03	1.03/4269.52	154.53/1015.34	15.19/615.97	_
N,N-Dimethylformamide	2735.77/25,896.8	11,638.91/11,215.14	45.69/2725.82	0.82/1839.0	94.13/514.64	12.45/390.5	_
Dimethylcyanamide	4767.25 ^b^	222.44 ^b^	732.57 ^b^	369.28 ^b^	125.20 ^b^	123.66 ^b^	202.93
1,1,4,4-Tetramethyl-2-tetrazene	1421.32 ^b^	715.35 ^b^	323.64 ^b^	120.51 ^b^	49.87 ^b^	64.79 ^b^	275.88

Notes: a, acute toxicity; b, toxicity baseline; c, chronic toxicity.

## Data Availability

Data will be made available on request.
